# Biomimetic hydrogel scaffolds for stimulating fibrotic responses: development of an in-vitro assay for implant material testing

**DOI:** 10.3389/fbioe.2025.1628630

**Published:** 2025-08-29

**Authors:** VJ Spoddig, Rasika S. Murkar, Sascha Kopp, Heike Walles

**Affiliations:** ^1^ Core Facility Tissue Engineering, Otto-von-Guericke University Magdeburg, Magdeburg, Germany; ^2^ Otto von Guericke Universität – Magdeburg, Experimentelle Thoraxchirurgie/Core Facility Tissue Engineering, Magdeburg, Germany

**Keywords:** foreign body reaction, tissue engineering, ceramic particles, titanium particles, steel particles, fibroblasts, macrophages, cytokine profiling, 3D collagen hydrogel

## Abstract

**Introduction:**

The foreign body response (FBR) is a complex immune response that affects implant integration and function. Conventional *in vivo* models are limited by ethical and reproducibility issues, emphasising the necessity for reliable *in vitro* alternatives. The objective of this study was to develop a standardised *in vitro* test using a collagen hydrogel-based 3D co-culture system to simulate FBR.

**Methodology:**

A 3D hydrogel model was co-cultured with human fibroblasts and macrophages to investigate immune responses to implant materials such as ceramic, titanium and steel. Cytokine expression and ECM remodelling were measured over a 14-day period to characterise material-specific responses.

**Results:**

The hydrogel model enabled a detailed analysis of the immune response to different materials. The material with the strongest fibrotic response was titanium, which resulted in a notable increase in collagen and TGF-β1 in M2 macrophage cultures. Furthermore, the emergence of IL-6 and IL-4 as prominent cytokine trends provided valuable insight into the inflammatory and regenerative response.

**Discussion:**

The model demonstrates that titanium exhibits a probable propensity for fibrosis, a finding that is corroborated by elevated TGF-β1 levels. IL-6 has been identified as a significant marker for inflammatory reactions. The results offer new perspectives for the development of patient-specific models, and future studies should include the comparison of fibroblasts from patients who have responded to implants with those who have not.

**Conclusion:**

The 3D hydrogel model offers a promising, cost-effective *in vitro* alternative for studying FBR and allows for a more accurate analysis of immune responses to implants. Future studies should further investigate the interactions of fibroblasts and macrophages and compare the immune responses between different patient groups to better understand the mechanisms behind different responses.

## 1 Introduction

**FIGURE 1:**
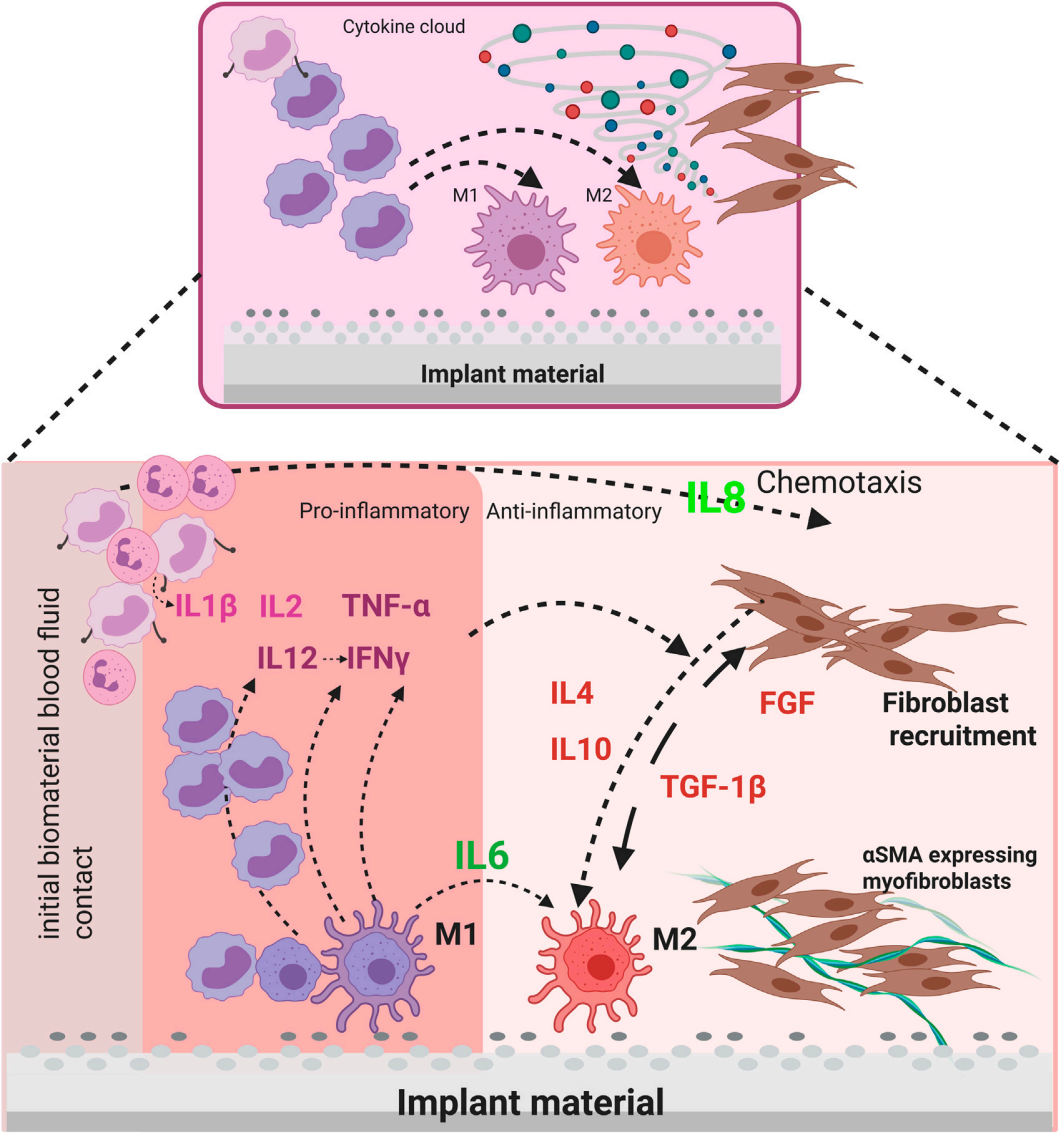
Schematic representation of the interaction between stromal cells (pFb) and immune cells (M1 & M2) via cytokines in the context of a foreign body reaction to implant material.

Advancements in biomimetic artificial scaffolds have created new opportunities in tissue engineering. Many of which work around the challenge of customizing substitutes for natural extracellular matrix (ECM) by utilizing different sources as well as manufacturing technologies. Collagen hydrogels are one among the most widely applied biomimetic scaffold materials in tissue engineering. Apart from providing the advantage of 3D structure similar to *in vivo*, due to their biocompatibility, ability to mimic the extracellular matrix 3D environment they do provide ease of functionalization and hydrogel matrix alone have proven the potential for vast range of tissue engineering applications. Clinical research on reliable and biocompatible materials has been a long tradition to improve the inventory for biomedical implants and to make it more clinically accepted in various operative procedures. Ceramic and metal implants are commonly employed in surgical pro-cedures, particularly in orthopaedic and thoracic surgery (Abdel et al., 2018). For designing assays aimed at testing implant reactions, biomimetic scaffolds offer an innovative platform. One such study demonstrates the promising approach of using pure collagen scaffolds for producing cell embedded structures with the help of modified 3D printing technology. Here the researchers used hMSCs embedded in the collagen, effectively demonstrated the collagen scaffolds to support cell adhesion, proliferation, and morphology (Palladino et al., 2024). Major challenge in order to develop an *in-vitro* foreign body assay to test biomaterials is the complexity of the fibrotic response. This consists of a cascade of reactions ranging from acute pro inflammatory stage towards fibrous encapsulation making it challenging to model *in vitro*. The response is influenced by the actions of two key cell types: fibroblasts and macrophages (Jannasch et al., 2017b). These cell types are involved in the regulation of inflammatory processes, tissue remodelling and fibrosis. Particles generated *in vivo* by mechanical and corrosive stress on implants are released into the tissue microenvironment. Surrounding cells, such as fibroblasts, begin to absorb the foreign bodies and activate the immune system via cytokines. Local macrophages are also activated (Bertrand et al., 2018). The activated macrophages initiate a cascade of inflammatory responses by releasing cytokines and chemokines (Bertrand et al., 2018). This process is known as the Foreign body response (FBR). The cytokines involved are shown in Figure 1. The FBR observed in the context of thoracic surgery and orthopaedics exhibit notable differences, particularly with respect to the localisation and composition of the affected tissues and the underlying biological mechanisms. In the context of thoracic surgery, foreign bodies such as implants, wires, or stents frequently elicit inflammatory responses in lung tissue, which may potentially give rise to fibrosis or infection. Such reactions are frequently characterised by mechanical stress and proximity to sensitive organs, including the heart and lungs (Anderson and Jiang, 2017; Liang et al., 2023). The symptoms may include respiratory distress or a localised infection. Whereas, FBR in orthopaedics are typically limited to bone and soft tissue. In this context, granulation tissue and fibrosis frequently develop around the implant, which can impede healing or even result in implant rejection. This can lead to persistent pain, erythema, swelling, osteolysis, and implant loosening (Bertrand et al., 2018; Gibon et al., 2024). It is important to note that the two disciplines have dissimilar therapeutic approaches to controlling inflammation and favouring healing, with orthopaedics often placing greater value on the mechanical stability of the implant.

The high biocompatibility and corrosion resistance of ceramic implants make them suitable for use in the fields of tissue engineering and regenerative medicine. This is due to the fact that they are less likely to elicit an immune response in the recipient of the implant. However, they can also elicit allergic reactions or intolerances in patients with highly sensitive immune systems, which underscores the necessity for a more profound comprehension of the interactions between biomaterials and living tissues (Swalsky et al., 2024). Metal implants, for example, those made of titanium or cobalt-chromium alloys, offer high strength and durability as a material. However, they are also known to cause allergic reactions or metal-related inflammation (Rae, 1981; Hanawa, 2022). This necessitates the integration of advancements in biomedical and pharmaceutical technology to optimize material selection. It is therefore imperative that allergological tests are conducted prior to implantation, and that specific material tests are carried out, in order to ascertain whether the patient exhibits any signs of hypersensitivity to the material in question (Hallab and Jacobs, 2009). In the standardised epicutaneous patch test, for instance, there is a potential risk of sensitisation that can be caused by the application of the foreign material to the skin. However, in the dermal fibroblast isolation procedure, this risk is eliminated due to the advanced preparation of the test subject (Agrup, 1968). Moreover it is frequently challenging to undertake an objective evaluation, as irritant and allergic reactions may exhibit phenotypical similarities. This can necessitate repeated testing, incur-ring additional costs and requiring additional resources (White, 2012). Furthermore, studies have demonstrated that the performance and assessment of the patch test are person dependent (Mowad, 2006). The performance of preoperative tests has been demonstrated to reduce the occurrence of postoperative complications and increase the success rate of implantation. The purpose of this study is to optimise the preparation and performance of biomaterials in clinical applications.

The objective of this study is to develop an *in vitro* assay that simulates the foreign body response (FBR) to the abrasion of implant materials. The abrasion described above occurs *in vivo* due to corrosive processes on the implant (Duffo et al., 1999; Mombelli et al., 2018). It is also caused by mechanical stress, for example, from respiratory movements. Depending on their physical constitution, adults breathe 12 to 20 times per minute during the whole day (Chourpiliadis and Bhardwaj, 2025). Chest implants are therefore exposed to continuous stress, which releases microparticles. This is a crucial aspect of tissue engineering and regenerative medicine. The hypothesis is that different particle types and sizes lead to significantly different cell and cytokine responses, providing insights into the pro- and anti-inflammatory and tissue regeneration pathways activated during FBR. The findings of this study have the potential to facilitate advancements in the domain of nanomedicine, particularly in the optimisation of implant materials to mitigate immune responses and promote healing. This research contributes to the development of innovative methods to restore form and function in the human body by exploring the interactions between biomaterials and human tissue. Jannasch et al. (2017a) have demonstrated that it is rational to store primary fibroblasts in a biomimetic 3D environment. Utilising the fibrin network, which is naturally formed as a result of haemostasis, as a 3D matrix, their approach offers a novel method for studying the behaviour of primary fibroblasts. In our *in vitro* assay, fibroblasts are stored in a 3D matrix consisting of a rat tail collagen-hydrogel mixture, enabling analysis of collagen remodelling and the behaviour of the fibroblasts. Furthermore, a two-chamber system has been developed to spatially separate macrophages and primary fibroblasts, facilitating comprehensive analysis. The communication between the two cell types is ensured by a semi-permeable membrane between the two chambers via cytokine interaction.

## 2 Materials and methods

### 2.1 Production and characterization of implant particles

Two ceramic particle variants, namely, CT800 and TMDAR were kindly provided by the Experimental Orthopedics department, Otto-von-Guericke University Hospital Magdeburg, Germany. Both the ceramic particle in powder form was weighted and adjusted to 10 mg and dissolved in 1 mL sterile distil water to obtain stock solution of 10 mg/mL concentration, which was used to prepare further dilutions - 1 and 10 μg/mL - stored at 4°C until further use.

Titanium (TIT) and steel (STE) particles were produced using established production process for generation of abrasion particles. The titanium and steel rods under consideration were obtained from MedXpert GmbH, Eschbach for the purposes of this investigation. Briefly, steel and titanium rods are fixed in allocated positions in a magnetic holder (3.5 cm), which is fixed without glue to avoid contamination. The holder is placed in a glass container (125 mL) with 70% distilled water and rotated on a magnetic heating plate. This process takes 7–10 days for titanium and 14–20 days for steel particle generation. After abrasion, the particles are sterilized by autoclaving to prevent contamination. The particles are then weighed, centrifuged and the supernatant removed. Finally, the weight of the particles is determined, and they are adjusted to the desired concentration in sterile PBS.

The particle size is determined by measuring Mie and Fraunhofer scattering. Mie scattering describes the scattering of light by particles that are larger than the wavelength of the incident light, while Fraunhofer scattering is used for smaller particles. The size distribution of the metal particles is demonstrated graphically in Supplementary Figure S1. Both types of scattering are based on different mathematical models (Kokhanovsky, 2008). For measurement the particles are suspended in a dispersing liquid, such as deionised water or pure ethanol. The following table summarized the used reference biomaterials including ceramic particles obtained from THA implants CT800 as well as steel and titanium particles (Table 1).

**TABLE 1:** Materials used and corresponding particle sizes (CT800, titanium and stainless steel).

Material	Description
CT800	Calcined alumina (Al2O3); Primary crystal size 1.9 µm; Unground; Fine controlled particle size distribution
Titanium	Size ranged from 0.7 to 80 µm with a frequency maximum 3 µm
Steel	Size ranged from 0.4 to 100 µm with two frequency maxima 8 and 69 µm

### 2.2 Cellular and biological components in cell culture

#### 2.2.1 General cell culture conditions

The cells were cultured in a controlled condition in an incubator with 5% CO_2_ at 37°C. The best cell growth can be obtained with a controlled cell culture medium with 10% serum supplement and antibiotics (Penicillin + Streptomycin, Sigma-Aldrich, Taufkirchen, Germany, P078) to prevent bacterial growth. All primary cell isolation and cultures were performed under the approval of the Local Ethics Committee of the University of Wuerzburg (182/10) and informed consent of the patients. The culture steps were also recorded. Cells were monitored regularly under the microscope. All the processes including medium exchange, passaging, assays were performed under sterile conditions using sterile cell specific mediums and supplements inside Biosafety cabinet. The cells were passaged when they have reached 90% confluency.

NIH3T3 (ATCC CRL1658, Germany) is known to be as standard fibroblast cell line, was kindly provided by the “Experimental orthopedic research lab, at University Hospital Magdeburg”. NIH3T3 is an adherent fibroblastic cell line, most commonly used in tissue engineering research. These were cultured starting from passage 2 and cultured in T175 flasks. NIH3T3 fibroblasts are then used in preliminary investigation of 2D fibroblast stimulation assay on a 12 well plate, to investigate particle uptake and RTPCR analysis. Briefly, fibroblast stimulation assay was performed in 12 well plate with NIH3T3 1 × 10^5^ cells per well with 2 mL fibroblast medium. After 24 h cells achieved 80%–90% confluence and were then treated overnight with particle dilutions (1, 10, 100 μg/mL) prepared in fibroblast medium under sterile conditions. CT800 ceramic Al_2_O_3_ particle (powder) were kindly provided by Experimental Orthopedic department, OvGU Clinic Magdeburg. After 24 h, cells were used in further standard processes of RNA isolation with Tryzol and RTPCR analysis, using lab standardized SOPs. The biological replications were performed using progressive passage numbers of NIH3T3 fibroblasts three times followed by consecutive RTPCR analysis with two technical replications. Similarly, phalloidin and DAPI staining’s were performed on the stimulated cells fixed on coverslips in 48 well plate.

Primary human dermal fibroblasts (pFb) were obtained from Chair of TERM, University Hospital Wuerzburg, Germany. These were isolated and used in culture under the approval of Local Ehical Committee of the University of Wuerzburg (182/10) and informed con-sent of the patients. In the study, isolated primary fibroblasts from a single donor were utilised for all test series, with the objective of eliminating individual differences between multiple donors. The pFbs were defreezed and cultured until passage 6 using standard operating protocols for defreezing and passaging. For setting up the pFb-hydrogel models with stimulated fibroblasts, firstly the fibroblast stimulation was performed in 2D cell culture flasks. The pFbs were cultured for 3 days in T75 adherent cell culture flasks at a density of 5 × 10^5^ and then the flasks were incubated with the respective material particles diluted to a concentration of 10 μg/mL for CT800 and 1 μg/mL for stainless steel and titanium. For the other group of unstimulated fibroblasts in collagen hydrogel, the same patient fibroblasts were used cultured in separate T75 flask without any particle contact.

THP-1 monocyte like cell line is widely used as a model for monocytes in research technologies. The cells are cultured in suspension culture at a density of 3 – 5 × 10^5^ cells/mL or less in a fresh medium. THP-1 cells were initially derived from ECACC and kindly donated by the “Heart & Thoracic research lab at University hospital Magdeburg” (Murkar et al., 2024). The THP-1 cell line is a spontaneously immortalised monocyte-like cell line, derived from a patient with acute monocytic leukaemia, specifically from the peripheral blood of a one-year-old male (Bosshart and Heinzelmann, 2016). THP-1 monocytic cell line follows a suspension culture technique. The cells were cultured in a density of 5 × 10^5^ cells/mL in a T175 cell culture flask and were controlled under microscope every third day. The cell culture can be maintained by the addition of fresh medium or by replacement of the medium. The cells are passaged when the cell confluence reaches almost 90%. The cell concentration is not allowed to seed above 1 × 10^6^ cells/mL. Subsequent cultures can be established with following resuspension at 2 – 4 × 10^5^ viable cells/mL. The media is changed every 2–3 days. While passaging, the cell suspension is centrifuged at 1,000 rpm for 5 min. Freezing protocol for the cells follow the same steps as in for NIH3T3 cell line. Following Table 2 summarizes the cell/cell lines, along with their culture medium composition and necessary order information for transparency.

**TABLE 2:** The specific cell types utilized in conjunction with the designated culture medium composition.

Cell/cell line	Medium composition
NIH3T3 (ATCC CRL1658, Germany)	DMEM high glucose (Sigma Aldrich, D5796) + 10% FBS (SUPERIOR stabil ^®^ FBS.S 0615, Bio&Sell Feucht, Germany)
Primary human dermal fibroblasts (pFb)	DMEM high glucose (Sigma Aldrich, D5796) + 10% FBS (SUPERIOR stabil ^®^ FBS.S 0615, Bio&Sell Feucht, Germany)
THP1 (ATCC TIB-202, Germany)	RPMI-1640 (2 mM L Glutamine) + 10% FBS (SUPERIOR stabil ^®^ FBS.S 0615, Bio&Sell Feucht, Germany)

#### 2.2.2 Macrophage differentiation

As described in previous publications, THP1 monocytes were differentiated into M0, M1, and M2 macrophages (Smith et al., 2015; Murkar et al., 2024). Briefly, cells in a density of 1 × 10^5^ were seeded in each well of a 12-well plate with coverslips and incubated for 1 hour with a THP-1 medium consisting of RPMI-1640 with 2 mM of L-glutamine (Gibco, Darmstadt, Germany, 21875034) + 10% FCS (Bio&Sell, Feucht bei Nürnberg, Germany, FBS.S0615) + 1% PenStrep (Sigma, Taufkirchen, Germany, P0781). Phorbol-12-myristat-13-acetat (PMA, Sigma-Aldrich, Taufkirchen, Germany, P1585) with a working concentration of 50 ng/mL was added and incubated for 24 h. After 24 h, the PMA-containing medium was removed and the cells were washed with the THP-1 medium three times. Then, the THP-1 medium was added and incubated for 1 h. For differentiation in the M0, M1, and M2 macrophages, different mediums were used as follows: M0 (THP-1 medium), M1 (THP-1 medium + IFN-γ (50 ng/mL), Sigma-Aldrich, SRP3058) and M2 [THP-1 medium + IL-4 (Sigma-Aldrich, SRP4137) + IL-13 (Sigma-Aldrich, SRP3274) (each 25 ng/mL)]. Differentiation in the cells was achieved after 48 h (Smith et al., 2015).

### 2.3 3D invitro hydrogel models construction

To prepare the hydrogel as a matrix for the stimulated fibroblasts, rat tail collagen was used, and crosslinked by increasing the GNS (Gel-Neutralisation solution), obtained from the University Hospital of Würzburg, by shifting the pH value. This procedure has already been described in the literature (Jannasch et al., 2017a; Islam et al., 2021; Yu et al., 2024) and lab standardized SOP was used to reproduce hydrogel models. Changing the pH leads to cross-linking of the collagen structures by changing the ionic interactions between the molecules, thereby activating reactive groups, such as amino groups, which are capable of forming cross-links (Reddy et al., 2015).

### 2.4 Development of co-culture

**FIGURE 2:**
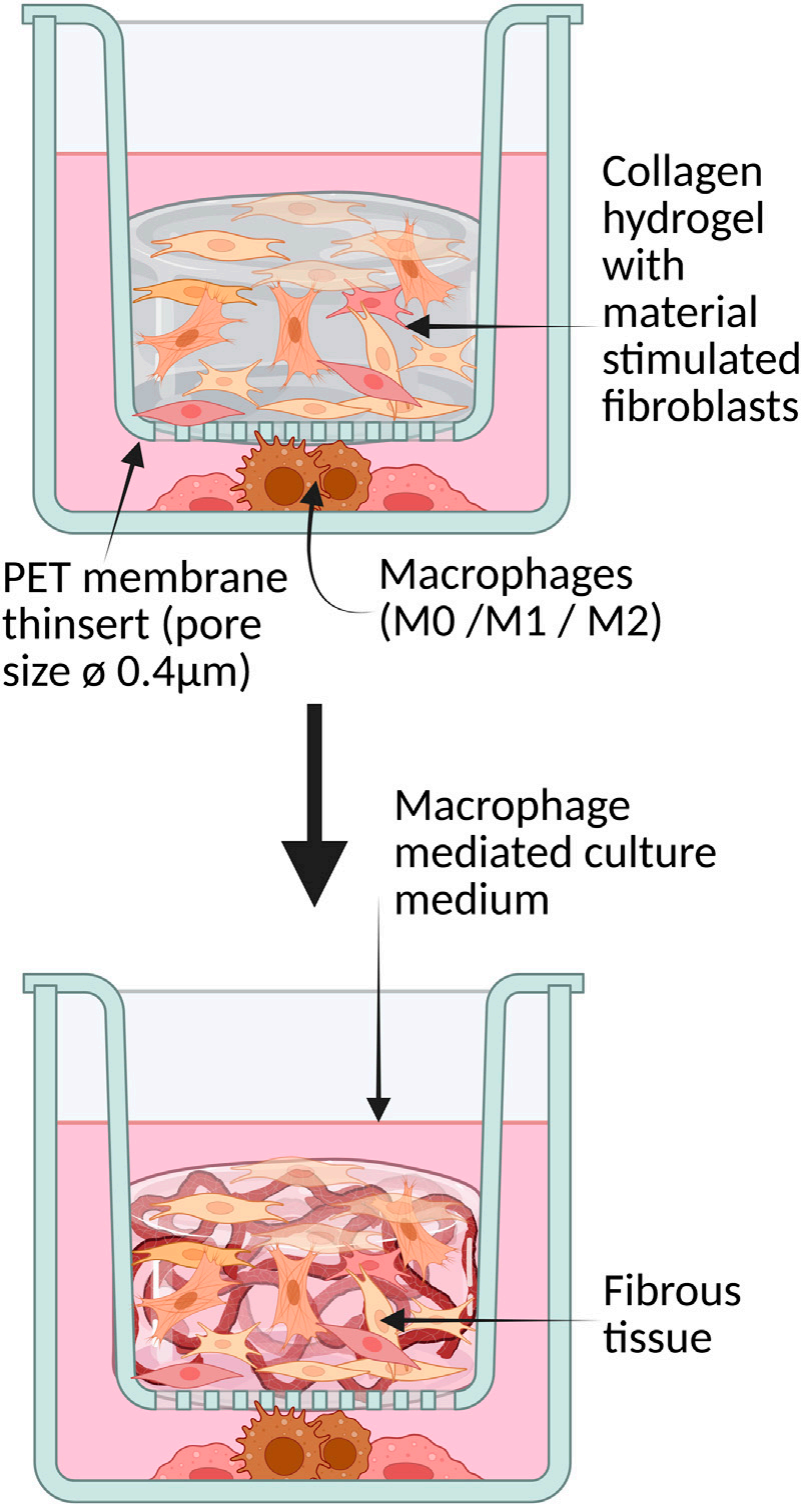
3D collagen hydrogel with primary fibroblasts in co-culture with macrophages separated by a semipermeable membrane.

As shown in the model (Figure 2), the 3D co-culture model was constructed using 3D collagen hydrogels prepared with fibroblasts stimulated by implant particles and the unstimulated group separately in two 12-well plates. The collagen hydrogels were then distributed in the 12-well inserts with semi-permeable membranes, while the macrophage subtypes were placed underneath inside the well. At the bottom of the well plate were 1 × 10^5^ macrophages of one type with 1.5 mL of THP1 medium. At the beginning of the culture and during the cultivation period, 500 µL of the fibroblast medium is added every 2 days to provide the necessary nutrients. The collagen hydrogels possess an initial volume of 1 mL and contain 1 × 10^5^ primary fibroblasts. The Trans-Well inserts were selected so that each point in the collagen matrix is no more than 1 cm from the surface, thereby ensuring sufficient supply to the cells by diffusion. This 3D model was maintained for 14 days and assessed at intervals (Day 3, 7, 10, 14). Macrophages were co-cultured with fibroblasts in 3D hydrogels to study their interactive effects over 14 days. The 3D collagen hydrogel culture was created on five occasions in total. However, contamination was observed in two experiments in which the extracellular matrix was generated by the fibroblasts. These experiments were therefore discarded, as it is to be expected that the cytokine production or FBR is altered by contamination, and therefore there is no comparability between the series of experiments. Hence only three biological repetitions are considered henceforth.

### 2.5 Characterization methodologies

#### 2.5.1 Immune-histological characterization

Analysis was performed using Super Vision 2 HRP Kit (DCS, Hamburg, Germany, PD000KIT) according to manufacturer protocols. The tissue sections were stained against actin, procollagen, fibronectin, collage1, Ki-67 in optimized concentrations as summarized in the table below along with corresponding negative controls Mouse IgG serum (Sigma-Aldrich, Taufkirchen, Germany, I8765) and rabbit IgG serum (Sigma-Aldrich, Taufkirchen, Germany, I8140). Previously established standard operating protocol for immunehistology was used (Murkar et al., 2024).

#### 2.5.2 Immunofluorescence characterization

Staining was performed according to the previously published protocols (Murkar et al., 2024) for macrophage characterization using the respective CD markers for M0, M1, and M2 macrophages, as shown in Table 2, as well as for cellular tissue characterization on the fixed *in vitro* tissue models. THP1 derived M0, M1, M2 macrophage populations were characterized using selected macrophage specific cell surface markers. The cell populations were differentiated in 12 well plate separately as explained in the methods section above and characterized using immunofluorescence staining according to the standardized protocols (20). Briefly, the cells were gently washed with PBS- and fixed in Paraformaldehyde (PanReac AppliChem, Darmstadt, Germany, 256462) for 10 min, which was followed by permeabilization (0.5% Tween20 in PBS-) and blocking steps with intermittent washing with PBS-. Following 1 h of blocking at room temperature, cells were coated with the respective primary antibodies (see Table 3) and counterstained with host-specific fluorescently labelled secondary antibodies and DAPI nuclei staining. Fluorescence imaging was performed using a ZEISS Axio Observer fluorescence microscope (Keyence GmbH, Neu-Isenburg, Germany).

**TABLE 3:** The following table provides a comprehensive overview of the antibodies utilized in this study, including the incubation time, temperature, and dilution ratio for each antibody.

Antibody	Manufacturer	Incubation time/temperature	Dilution IHC	Dilution IF	Cells positive
mCD68	Invitrogen, Darmstadt, Germany 14-0688-82	Overnight, 4°C	1:500	5 μg/mL	M0
rCD80	Invitrogen, Darmstadt, Germany PA585913	Overnight, 4°C	1:500	1:100	M1
rCD163	BIOSUSA, Massachusetts United States bsm-54015R	Overnight, 4°C	1:100	1:100	M2
Anti-Actin	Sigma Aldrich, Taufkirchen, Germany, A2103	1 h, room tem-perature	1:1,000	1:1,000	—
Anti-Fibronectin	Invitrogen, Darmstadt, Germany, MA5-11981	1 h, room tem-perature	1:100		—
Anti-Procollagen	Emd Millipore, Massachusetts, United States, 3468667	1 h, room tem-perature	1:50	—	—
Anti-Ki67	Sigma Aldrich, Taufkirchen, Germany, Q3194397	1 h, room tem-perature	1:200	—	—
Anti-Collagen1	Sigma Aldrich, Taufkirchen, Germany, C2456	1 h, room tem-perature	1:2,000	—	—
Anti-mouse IgG FITC	Sigma Aldrich, Taufkirchen, Germany, F0257	1 h, room temperature	—	1:50	—
Anti-rabbit IgG (H + L)	Sigma Aldrich, Taufkirchen, Germany, SAB4600084	1 h, room temperature	—	10 μg/mL	—
DAPI (Fluoromount-G)	Invitrogen, Darmstadt, Germany 00-4959-52	—	—	1 drop/slide	—

The tissue sections were also stained against primary antibodies specifically fibronectin, Procollagen, Ki-67 along with DAPI nuclear counterstain in concentrations as summarized in the table below. According to the standardized protocols (Murkar et al., 2024), briefly, after blocking using 5% FCS + 1% BSA in PBS- for 4 h, these primary antibodies (for incubation time, refer to Table 3) were counterstained using host-specific fluorescently labelled secondary antibodies. Finally, nuclei were stained blue using DAPI.

#### 2.5.3 Functional characterization using sandwich and multiplex ELISA

Supernatants from 3D co-cultures were collected and analyzed first using Sandwich ELISA for IL-6 and TNF-α. The measurement of cytokines IL-6 and TNF-α is a practicability item for the valuation of foreign body tissue response. These cytokines have a key role in the inflammatory response and are essential mediators of the immune response to implants. A multiplex enzyme-linked immunosorbent assay (ELISA) was conducted using the LEGENDplex essential immunoresponse panel to quantify the levels of nine cytokine markers (Supplementary Table S1). Supernatants were collected from stimulated primary human fibroblasts cultured in 3D collagen hydrogels in co-culture with M1 and M2 macrophages on days 3, 7, and 10 for ceramics, as well as on day 14 after stimulation with steel and titanium. All samples were collected under sterile conditions. Subsequently, the samples were stored at −80°C in a sterile state until measurement was undertaken using the multiplex ELISA method. The multiplex ELISA sub-panel mix-and-match kit permitted the measurement of the expression of IL-1β, IL-2, IL-4 IL-6, IL-8, IL-10, IL-12, TNF-α and TGF-β1 in a multiplex setting using the flow cytometer FACS CantoII FACS machine. The results were subsequently analysed using the BioLEGEND software, which is available online from the panel manufacturer.

#### 2.5.4 Graphical evaluation of the immunohistological results

Image processing was done using the Image Processing Toolbox™ (The MathWorks, Inc. (2022). Image Processing Toolbox version: 11.5 (R2022a), n.d.) in MATLAB R2022a. Images were processed using a semi-supervised algorithm where the raw RGB images were converted into HSV images (Supplementary Figure S2). Segmentation of the area corresponding to the tissue was done on the hue channel of the HSV image by using an automated global threshold using Otsu’s method (Otsu, 1979). Identification of the area within the tissue which was positively stained, was done using a fixed threshold on the saturation channel of the HSV image. The output images were individually inspected to ensure a successful segmentation and identification process. The percentage of tissue stained positively was then defined as follows:

$$\%\;of\;IHC\;positive\;tissue=\frac{IHC\;positive\;area\;within\;the\;tissue\;\left(Pixels\right)}{Area\;of\;the\;tissue\;\left(Pixels\right)}\times 100$$

#### 2.5.5 qRT-PCR for 2D fibroblast stimulation assay

In the case of 2D fibroblast culture of NIH3T3 fibroblasts stimulated with CT800 ceramic particles, RNA isolation approach was followed using the trizol lysing method according to the standard operating protocol for “RNA isolation”. Henceforth followed by reverse transcription to obtain copy DNA for PCR analysis of specific target genes, namely, GAPDH, TNF-α, TGF-ß1, IL-1ß, FGF, collagen-1 (Col-1), fibronectin (FN), IL-6. Table 4 shows gene primer sequences for selected genes. RTPCR analysis was repeated for three biological replications of NIH3T3 ceramic particle stimulation, along with each three technical replicates.

**TABLE 4:** RT-PCR target gene primers sequences used in PCR reaction.

Gene	Forward primer	Reverse primer
GAPDH	AGC AAG GAC ACT GAG CAA GAG AGG	GGG TCT GGG ATG GAA ATT GTG AGG
FN1	CGG​TTT​CCC​ATT​ACG​CCA​TT	CAC​GTT​GCT​TCA​TGG​GGA​TCA
α-SMA	CTA​TTC​CTT​CGT​GAC​TAC​TGC​CGA​G	GCT​GTT​ATA​GGT​GGT​TTC​GTG​GA
IL-6	CTC TGG GAA ATC GTG GAA A	CCA GTT TGG TAG CAT CCA TC
TGF-1ß	ACC​GCA​ACA​ACG​CCA​TCT​AT	TGC​CGT​ACA​ACT​CCA​GTG​AC
FGF1	GGA​GCG​ACC​AGC​ACA​TTC​AG	GCG​AGC​CGT​ATA​AAA​GCC​CT
Coll1	GGA​GAG​AGC​ATG​ACC​GAT​GG	AAG​TTC​CGG​TGT​GAC​TCG​TG
TNF-α	GGG CAG GTC TAC TTT GGA GTC ATT ATT	CTG AGA CAG AGG CAA CCT GAC

#### 2.5.6 Statistical analysis

Quantitative RT-PCR data was collected from three experimental repetitions and three technical replicates for each model. Average of quantity mean (q-mean) was obtained for each replicates and normalized against home gene GAPDH. Fold change was then calculated using the values from control samples where no CT800 stimulation was performed against those stimulated with controlled concentrations of CT800, namely, 1, 10, 100 μg/mL. Finally, the fold change data obtained for each experimental replicates is averaged and depicted in the supplementary section of this article. Additional statistical analysis was performed using OriginPro 2019 software, by pooling the raw data for RTPCR. Briefly, Friedman ANOVA test was performed on the calculated normalized q mean values and p values were calculated. To analyse the results of the ELISA measurements, three series of tests were conducted. In each independent test series, one sample was taken from each material, sampling time point and macrophage population in the co-culture. Each sample was analysed twice for the respective cytokine concentration, and the resulting values were averaged to form a mean value. This mean value was then combined with the respective mean values of the other two test series to form a general mean value. In this way, we achieved a test size of n = 6 for each test group. The Microsoft Excel programme was utilised for this process, as well as for the graphical representation of the mean value (Supplementary Figures S3A–F, S5A–F). The results were visualised in bar charts showing the measured concentration (pg/mL) of the different cytokines over time from day 3 to day 10 (A-B) or day 14 (Supplementary Figures S3A–F, S5C–F).

## 3 Results

### 3.1 2D particle uptake and fibroblast activation assay

**FIGURE 3:**
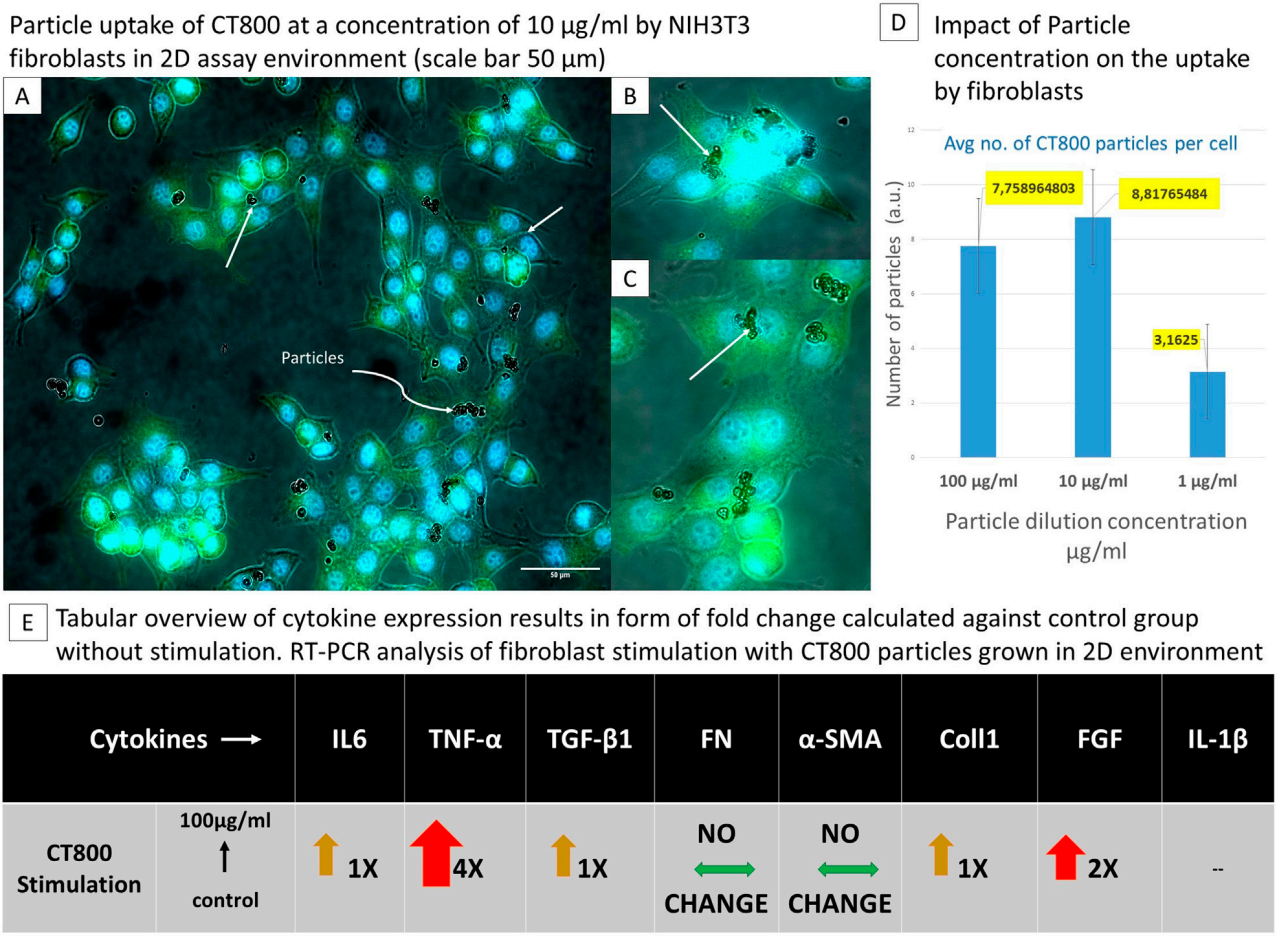
The tabular representation demonstrates the dependence of cell behaviour on particle size following particle stimulation in two dimensions. [Top left panel: **(A–C)**] Fibroblasts uptake of CT800 particles depicted using cells cytoplasma stained with phalloiding in fluorescent green and nucleus with DAPI in fluorescent blue, white arrows specifically indicate cells with particles, **(B, C)** are the emphasized image 6× fold of the par-ticle uptake of a colony of cells. [Top Right panel: **(D)**] Comparison of average number of particles uptake by one fibroblast. [Bottom horizontal panel: **(E)**] Tabular overview of cytokine expression from RT-PCR analysis of 2D fibroblast particle stimulation.

A 2D *in vitro* fibroblast stimulation assay demonstrated that ceramic particles (CT800) were actively internalised by the murine fibroblast cell line NIH3T3, as evidenced by the use of phalloidin and DAPI staining (Figures 3A–D). Figure 1 below depicts the uptake of particles. The stimulation of fibroblasts was conducted using NIH3T3 cells stimulated with ceramic particles CT800 at three distinct concentrations: 100, 10, and 1 μg/mL. Figures 3A–C illustrate the cell cytoplasm in green, stained by phalloidin, and the nucleus in blue, stained by DAPI. Furthermore, RNA analysis using quantitative real-time polymerase chain reaction (qRT-PCR) confirmed the presence of cytokine expressions associated with the inflammatory and anti-inflammatory micro-environments generated as a result of the FBR, which consisted of tumour necrosis factor alpha (TNF-α), transforming growth factor beta 1 (TGF-β1), interleukin 1 beta (IL-1β), interleukin 6 (IL-6), fibroblast growth factor (FGF), alpha-smooth muscle actin (α-SMA), type I collagen (COL1), and fibronectin [alongside glyceraldehyde-3-phosphate dehydrogenase (GAPDH) as a house-keeping gene]. The uptake of particles and the number of fibroblasts containing particles were quantified using Fiji software, and graphs were generated. It is evident that less than 50% of the fibroblasts have taken up the CT800 particles. The white arrows in the image indicate the presence of particles within cells. Figure 3B is a magnified image (×6) of the particle uptake of a colony of cells, while Figure 3C shows a cell with CT800 particles within its cytoplasm. The number of cells and particles taken up by each cell was determined using image analysis software. Following the enumeration of the total number of cells in each of the four sample images, which were taken of each group [i.e., CT800 (100 μg/mL), CT800 (10 μg/mL) and CT800 (1 μg/mL), and the control without particle stimulation], the number of particles taken up by each cell was also counted.

**FIGURE 4:**
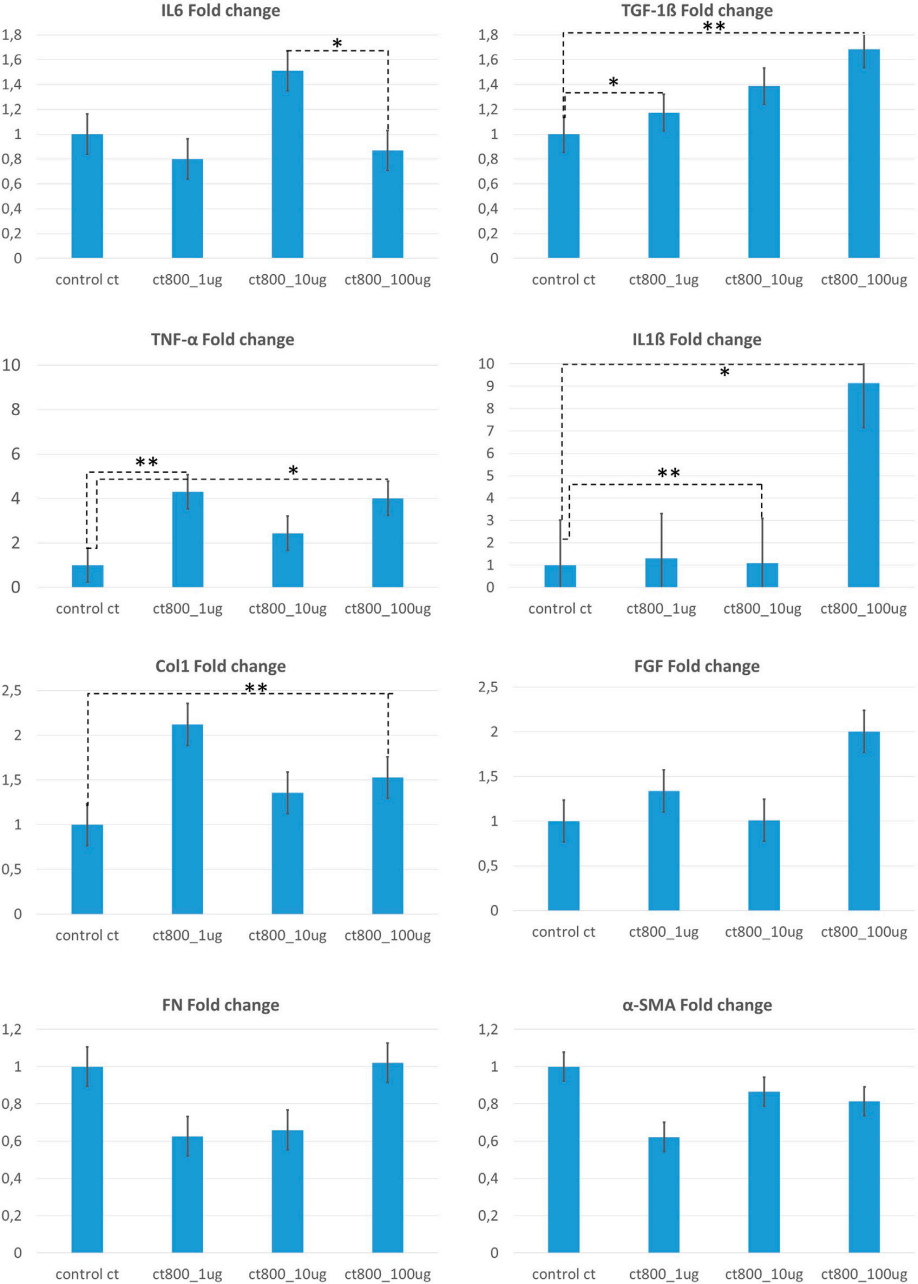
Summarized fold change analysis for gene expression from 2D fibroblast stimulation assay using CT800 particles in concentrations of 100μg/ml, 10 μg/ml and 1 μg/ml. Fold change was calculated with respect to the control group (un-stimulated fibroblasts). Above figure shows fold change analysis for expression of IL6, TNF-α, Col1, FN, TGF-1β, IL1β, FGF, α-SMA. [Statistical analysis performed using Origin Pro 2019 software with non-parametric Friedmann ANOVA tests * p<0.05; ** p<0.02].

In conclusion, the data demonstrate that ceramic particles are effectively uptaken by NIH3T3 fibroblasts, with dose-dependent uptake quantified. The RT-PCR results indicated that exposure to particles resulted in the activation of fibroblasts as shown in Figure 4. The enhanced up-take of particles was observed to result from the nanometer-sized particles, whilst also giving rise to increased cell mortality. The enhanced inflammatory response towards such particle uptake was substantiated with the expression of 6 fold increased TNF-α in the case of stimulation of fibroblasts with 100 μg/mL of TMDAR particles. Whilst there was also enhanced expression of IL-6. In the case of CT800 stimulation, along with TNF-α.

### 3.2 Characterization of macrophage phenotype

Differentiation and immunofluorescence confirmed distinct M0, M1, and M2 macrophage populations, each characterized by specific markers. In *In-vitro* conditions, it is possible to polarize macrophages towards inflammatory phenotype M1 either by infectious microorganism-related molecules (e.g., the Gram-negative product LPS) or also by inflammation related cytokines TNF-α or IFN-γ, alone or in combination (Nathan et al., 1983). Meanwhile M2 polarization was optimized *in-vitro* by implementing Th2 related cytokines, namely, IL-4 or IL-13 (Locke et al., 2019; Peng et al., 2023). Such stimulation using IL-4, IL-13 is known to induce M2a subtype polarization of macrophages responsible mainly for the enhanced endocytic activity, promoting cell growth and tissue repair. To facilitate a precise and efficient differentiation of THP1 monocytes into their respective phenotypes, immunofluorescence imaging was performed. Briefly, specific surface markers, as outlined in Table 3, were stained to distinguish be-tween the phenotypes. The respective serum was substituted for the primary antibody to prevent non-specific binding of secondary antibodies. DAPI was used for counterstaining.

THP1-derived M0 macrophages showed positive staining for CD68, identified with a FITC secondary antibody, while CD80 and CD163, markers for M1 and M2 macrophages respectively, and showed no staining. M1 macro-phages exhibited positive staining for the CD80 marker. Interestingly, some M1 macrophages also stained positive for the CD163 marker, typically associated with M2 cells. M2 macrophages demonstrated positive staining for CD163, and a subset also stained positive for CD80, indicating a small population of M1 cells within the M2 group. Negative controls showed no staining for any secondary antibody. Additionally, M1 macrophages appeared more spindle shaped and elongated compared to the more circular M0 macrophages. When comparing figures for M0, M0 macrophages were visually smaller than M2 macrophages. The characterization was published successfully in (Murkar et al., 2024).

### 3.3 Dynamic changes in 3D collagen hydrogel monoculture models

**FIGURE 5:**
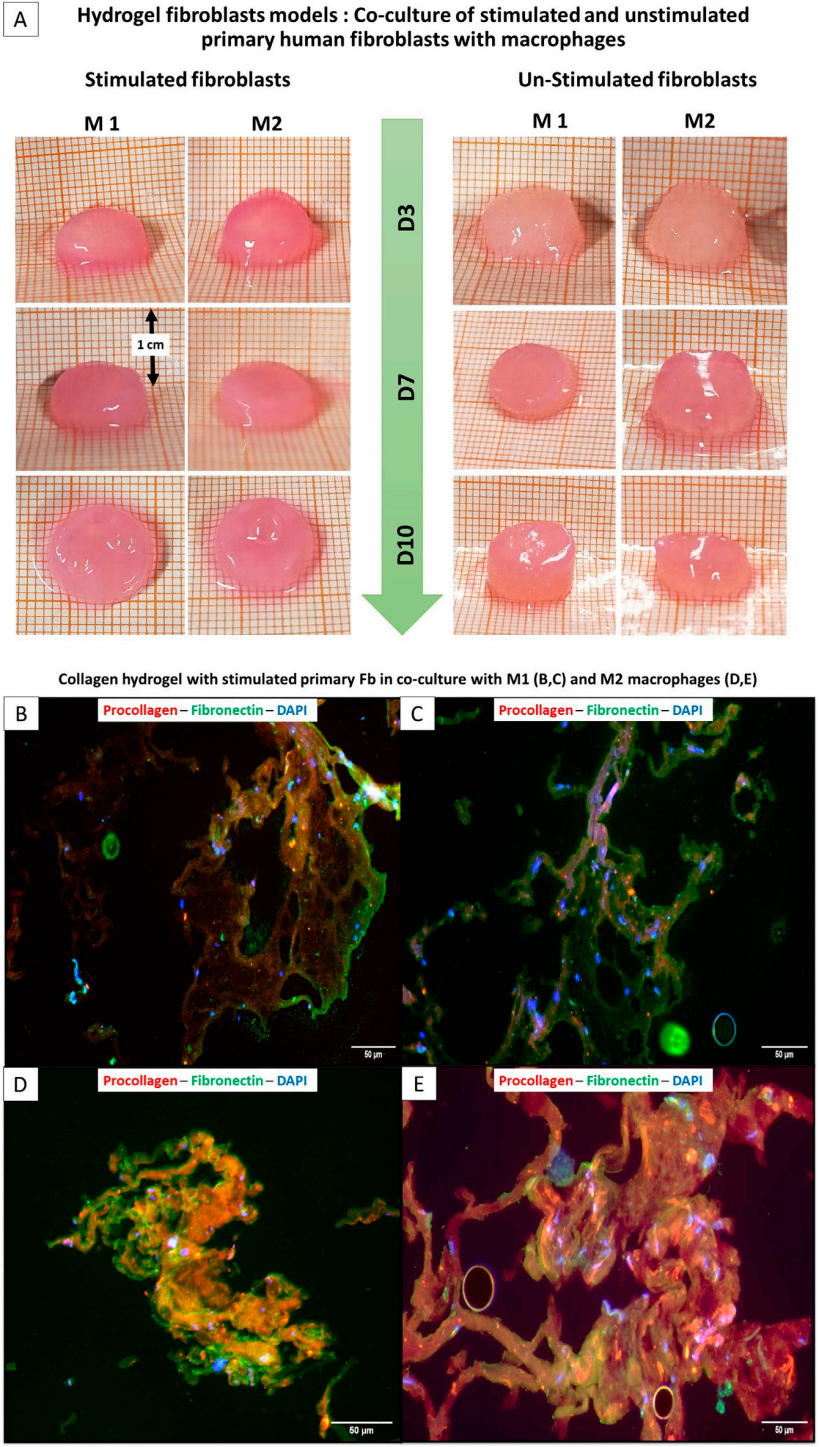
Comparison of the size of hydrogel fibroblasts Model: Stimulated and non-stimulated primary fibroblasts with M1 and M2 macrophages (Top panel: **A**) ; (Bottom panel) Immunoflurescence of collagen hydrogel-embedded stimulated primary fibroblasts with M1 (**B,C** (Top)) and M2 (**D,E** (bottom)).

**FIGURE 6:**
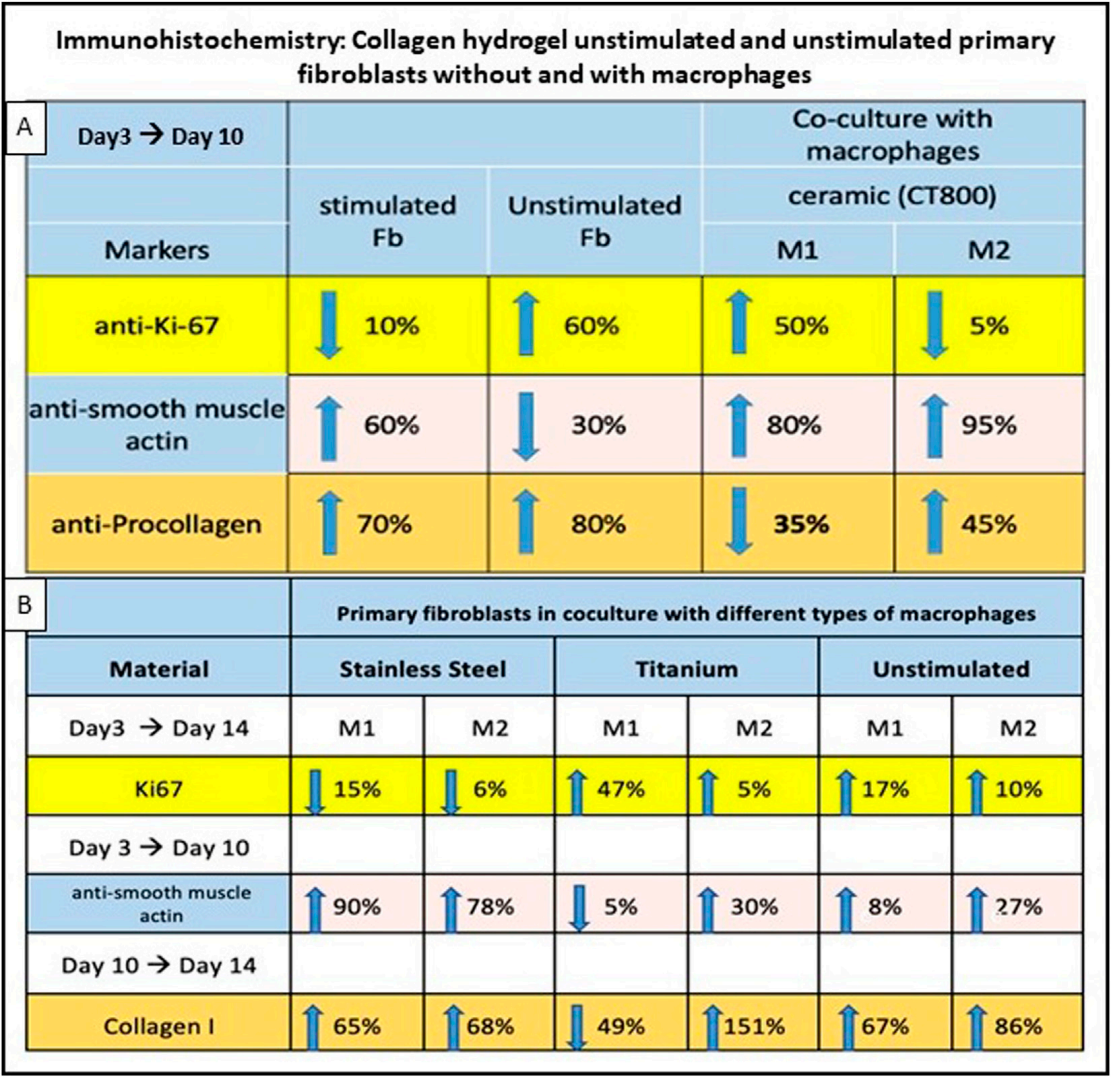
Immunohistochemistry results of collagen hydrogel with stimulated and unstimulated broblasts, with and without macrophages. **(A)** simulation with CT800, percentages for anti-Ki-67, anti-smooth muscle actin, and anti-procollagen markers from day 3 to day 10. **(B)** compares different materials (stainless steel, titanium, unstimulated) with macrophages over various days for Ki67, anti-smooth muscle actin, and Collagen I markers. Arrows indicate trends percentages are provided for each condition.

Fibroblasts embedded in the collagen hydrogel showed time-dependent changes in proliferation, fibronectin, and procollagen expression, reflecting cellular remodeling and matrix interaction as seen visually in Figure 5A. Collagen-hydrogel models embedded with pre-stimulated primary dermal fibroblasts with CT800 ceramic particles (100 μg/mL), cultured along 10 days were monitored on days 3, 7, 10 and fixed to perform immuno-histochemical analysis using selected cell surface antibodies indicative of motility, proliferation, and collagen production included anti-actin, anti-Ki-67, anti-procollagen, and anti-fibronectin, alongside mouse and rabbit IgG as negative controls. In the monoculture of stimulated fibroblasts within the collagen hydrogel, positive staining for actin indicated active cell motility, while Ki-67 confirmed cell proliferation. Staining intensity was quanti-fied on a percentile scale from 0% to 100%. Comparison of immunohistochemically stained sections from unstimulated versus stimulated fibroblasts showed that actin was 90% positive from day 3 to day 14, while Ki-67 expression decreased by day 14. Procollagen levels remained constant, as summarized in table form in Figure 6A. Consequently, co-cultures with differentiated macrophages and both stimulated and unstimulated fibro-blasts were established for the next phase of the study.

### 3.4 Macrophage-fibroblast interactions in co-culture

Co-culture studies revealed that macrophage phenotype significantly influences fibroblast behavior and matrix remodeling within the 3D hydrogel environment. As illustrated in Figure 2 above, co-culture was setup between THP-1 derived distinct populations of M1, M2 and M0 macrophages (in separate wells) in a 12 well plate and the fibroblast collagen hydrogels built in the permeable trans well inserts. Upon IHC stainings for the similar markers, it was clear that actin was stained positively showing the active participation of cells and motility. As summarized in Figure 6B below, in co-cultures of unstimulated fibroblasts with M1 versus M2 macrophages, collagen I was highly expressed on day 10 with M2 macrophages, along with increased Ki-67 expression. Similar to the cultures that were not stimulated or stimulated with ceramic, it was found that the cell proliferation marker Ki67 increased in titanium in both M1 and M2 or remained approximately the same, while it decreased in steel in both macrophage types between the 3rd and 14th day (Figure 6B). Procollagen was highly expressed (around 50%) on day 3 but decreased to approximately 35% by day 10 with M1 (Figure 6A). In contrast, the M2 culture exhibited a contrasting response to stimulation with CT800, demonstrating an increase of 45%. Immunofluorescence staining showed that green fluorescence indicating fibronectin was primarily located outside the cell borders, forming a layered structure, while red fluorescence marking procollagen was located within the cell membranes. In the co-culture of stimulated fibroblasts with M1 macrophages (Figures 5B,C), green fluorescence significantly marked the outer sheaths, whereas red fluorescence was reduced compared to the increased green signal. In contrast, in the co-culture with M2 macrophages (Figures 5D,E), red fluorescence increased, and green fluorescence from fibronectin de-creased but remained visible. In the unstimulated fibroblasts co-cultured with M1 macro-phages, fibronectin expression significantly increased while procollagen expression de-creased.

Since a longer period of 14 days was to be examined during stimulation with metal particles, collagen 1 was examined as the end product of fibroblast synthesis instead of the precursor molecule procollagen. It is noticeable that collagen synthesis increased on day 14 in all cultures with M2 macrophages (Figures 6A,B). This was most pronounced in titanium, where an increase of 151% was measured. This contrasts with a decrease in collagen synthesis in the M1 macrophages after titanium stimulation. The two macrophage types showed the clearest difference after contact with titanium particles. In the case of steel and the non-stimulated cultures, a comparatively small difference can be seen be-tween the macrophage groups [steel: ∆M2 (%)-M1 (%) = 3%/unst.: ∆M2 (%)-M1 (%) = 19%] In co-culture with M2 macrophages, Ki-67 expression dropped by about 5% by day 10. A notable distinction between the M1 and M2 macrophages can be observed in the procollagen expression (Figure 6A) for CT800 and collagen I expression (Figure 6B) for titanium. A significant reduction of 35% (CT800) and 49% (titanium) was observed in the M1 macrophages, while the proportion of M2 macrophages increased by 45% (CT800) and 151% (titanium). This also differentiates the two materials from stainless steel and the unstimulated culture, in which the trend is analogous for M1 and M2.

### 3.5 Cytokine response profiling

ELISA results demonstrated distinct cytokine profiles, correlating with the type of implant material and macrophage phenotype, providing insights into the inflammatory and regenerative pathways activated during FBR.

#### 3.5.1 Dynamic expression of IL-6 and TNF-α cytokines in stimulated co-cultures

The measurements for the prominent cytokines, TNF-α and IL-6, were performed using a sandwich enzyme-linked immunosorbent assay (ELISA). TNF-α concentrations exhibited minimal variation in the majority of cultures, reaching negligible levels. In each stimulated and unstimulated co-cultures, including M1 and M2 macrophages, TNF-α demonstrated a consistent concentration (Supplementary Figures S3A–F). The absolute values of TNF-α were found to be lower in the CT800 (Supplementary Figures S4A,B) concentration than in the un-stimulated or metal particle-stimulated cultures (Supplementary Figures S3A–F). Irrespective of the stimulation employed, the TNF-α level remained at a lower level than in the unstimulated culture and exhibited no discernible trend. As shown in Supplementary Figures S4A,B the cultures stimulated with ceramic exhibited an increase in IL-6 concentration over time up to day 10, with the concentration in the M2 culture being considerably higher than in the M1 culture [cM1 (ceramic) = 139 pg/mL; cM2 (ceramic) = 1,344.0 pg/mL]. Notably, distinct variations were evident in the IL-6 concentration of cultures subjected to stimulation with stain-less steel or titanium particles. In the cultures that had not been stimulated previously and which contained both M1 and M2 macrophages, no measurable concentration of IL-6 was observed in the supernatant, with the exception of the M1 cultures on day 14 (Supplementary Figures S3A,B). Following stimulation with stainless steel, both the M1 and M2 cultures exhibited an almost identical concentration between days 3 and 7. However, this increased on day 14 [cM1 (steel) = 48.1 pg/mL; cM2 (steel) = 63.1 pg/mL]. In contrast, the IL-6 concentration after stimulation with titanium particles exhibited an increase from day 3, with the initial concentrations being higher with titanium as with stainless steel [cM1 (steel) = 3.8 pg/mL < cM1 (titanium) = 23.7 pg/mL; cM2 (steel) = 20.5 pg/mL < cM2 (titanium) = 39.1 pg/mL]. Following stimulation with titanium particles, a difference of >20 pg/mL was noticed in the final measurement (day 14) [cM1 (titanium) = 49.4 pg/ml/cM2 (titanium) = 73.6 pg/mL]. According-ly, the M1 and M2 co-cultures on day 14 exhibited notable discrepancies in IL-6 concentration for both metal types (Supplementary Figures S3C–F).

#### 3.5.2 Dynamic expression of additional cytokines in co-cultures

A comparative analysis of cytokine concentrations was performed by multiplex ELISA after stimulation with CT800 (Supplementary Figures S4A,B), titanium (Supplementary Figures S4C,D) and stainless steel (Supplementary Figures S4E,F), in each case using M1 or M2 macrophages as a co-culture. For the stimulation with CT800, the analysis was conducted over a period of 10 days to determine the cytokines IL-10, TNF-α, IL-1β, IL-8 and TGF-β1. Following stimulation with metal particles, the period was extended to 14 days to measure IL-4, IL-2, IL-1β, IL-12 and TGF-β1. Significant differences were observed between M1 and M2 co-cultures following stimulation with CT800, particularly in the case of TGF-β1. The concentration of TGF-β1 in M1 macrophages exhibited a decline from day 3 to day 10 (Supplementary Figure S4A), whereas in the M2 culture, the TGF-β1 concentration in the cell supernatant demonstrated a marked increase *(c3d(CT800) = 7.7 pg/mL; c10d(CT800) = 1,372 pg/mL)*. The concentration of IL-8 in the M1 culture had a continuous increase, with a doubling of the concentration was observed between the 3rd and 10th day. In contrast, the M2 co-culture exhibited a markedly elevated initial concentration of IL-8 on day 3, which declined by a factor of 17 by day 7 and subsequently rebounded. However, the final concentration did not reach the initial level. No discernible difference or trend was identified in the concentration of the cytokines TNF-α, IL-1β and IL-10 Following stimulation with metal particles, a discernible pattern was observed in the production of TGF-β1. Upon stimulation with titanium in co-culture with M2 macrophages, a continuous increase in TGF-β1 concentration over time was observed (Supplementary Figure S4D), whereas in the M1 culture, the maximum concentration was reached on day 7 (Supplementary Figure S4C). Following stimulation with stainless steel, the TGF-β1 concentration in both co-cultures (M1 and M2) exhibited a comparable trajectory to that observed after titanium stimulation in the M1 culture, with no discernible continuous increase between the third and fourteenth days. Furthermore, the concentration of IL-4 demonstrated disparities between M1 and M2 macrophages. The expression of IL-4 was significantly higher in the M2 culture than in the M1 culture at the outset of the experiment (on day 3 and 7) following stimulation with both titanium and stainless steel. On day 3, IL-4 expression was approximately five-fold higher in the M2 culture following titanium stimulation, and on day 7, it was approximately tenfold higher than in the M1 culture. A comparable, albeit less pronounced, discrepancy was noted following stainless steel stimulation. Subsequently, IL-4 concentrations declined following both titanium and steel stimulation. No discernible trends in cytokine concentration were observed over the course of the analysis for IL-2, IL-1β, and IL-12.

## 4 Discussion

Over the past few years, research on biomimetic scaffolds have outgrown exponentially, allowing them to offer a structural and metabolic environment that tries to resemble native tissues. Collagen based hydrogels in particular depicted promising results with their capacity to reproduce the ECM like structures and promote cell adhesions, proliferation and differentiation (Jalalypour et al., 2016). This study delves into the simulation of complicated foreign body response involved during implantation processes like in the case of orthopaedic and thoracic implants, by employing collagen hydrogel based 3D *in vitro* models. The review article by Jiang et al., sheds light on the applicability of hybrid biomaterials incorporating ECM-mimicking properties to effectively simulate these reactions, offering valuable insights for implant testing (Jiang et al., 2021). A critical challenge in developing *in vitro* assays for foreign body reactions lies in accurately modeling the complex fibrotic response, which includes a sequence of inflammatory and encapsulation events.

### 4.1 Ideal *in vitro* biosystem with migration of immune cells

Foreign body reaction is a major concern that arises subsequent to the implantation of biomaterials into the human body (Jannasch et al., 2017b). Consequently, it is imperative to formulate an ideal *in vitro* model that closely resembles the structure and behaviour of the tissue *in vivo*. It is of the extraordinary importance to create a system that is not only highly clinically relevant but also practical, cost-effective, reliable and can be used within a short time frame to enable pre-surgical testing. The construction of such a model necessitates the incorporation of stromal cells, for example, fibroblasts, into a matrix that mimics the three-dimensional tissue structure observed in the human organism. This fundamental tenet is corroborated by recent studies which have demonstrated that the cellular response within a three-dimensional test structure differs from that observed in a two-dimensional environment (Migulina et al., 2024). The experimental setup constitutes an ideal test system to mimic the *in vivo* foreign body reaction. It requires human connective tissue cells (in this case, primary human fibroblasts of the skin) and immune cells that have the ability to phagocytose foreign particles (macrophages differentiated from THP1 cells). To mimic the native like ECM properties and components, a rat tail collagen hydrogel matrix was used in which the primary fibroblasts are incorporated. Mechanical properties such as stiffness and viscoelasticity are known to fundamentally affect fibroblast activity and responses, although we did not characterize them explicitly, the collagen-based 3D model used here follows a well-established protocol previously validated in multiple studies (Rossi et al., 2015; Jannasch et al., 2017a; Reuter et al., 2017; Müller et al., 2024). The supply of embedded cells is facilitated by diffusion, with superficial cells receiving greater levels of supply than cells embedded deep in the matrix (<1 cm from the surface). However, this diffusion gradient within the tissue also reflects the physiological *in vivo* situation, in which cells in a tissue are also supplied with nutrients or oxygen to varying degrees (Gurski et al., 2010).

The foreign body reaction was simulated by the contact of primary fibroblasts with the foreign material. In the initial phase of the experimental setup, the fibroblasts were stimulated with CT800 at varying particle concentrations (1, 10, 100 μg/mL) to ascertain the optimal dosage. These particle concentrations were selected based on preliminary RT-PCR screening in 2D-stimulated NIH3T3 fibroblasts, where these doses induced fibrotic gene expression without apparent cytotoxicity; this was further supported by phalloidin and DAPI staining, which confirmed maintained cell morphology and tolerable particle-to-cell ratios under these conditions. The induced reaction should be sufficiently robust to elicit a measurable tissue response while avoiding the potential for cytotoxicity due to excessive concentration. A suitable tissue reaction was observed at 10 μg/mL. However, when stimulating with metal particles (stainless steel and titanium), a concentration of 10 μg/mL was found to be too high, inducing apoptosis in the fibroblasts. Consequently, the dose was reduced to 1 μg/mL. This suggests that the concentration of particles for fibroblast stimulation must be adjusted depending on the foreign body material.

Macrophages are the second vital component driving the foreign body response *in vivo* (Anderson et al., 2008; Jannasch et al., 2017b). During an inflammatory response, such as a tissue reaction to an implant, immune cells, including macrophages, migrate into the affected tissue (Hörner et al., 2004) (34). In order to simulate an immune response to external stimuli, macrophages have been integrated into the co-culture model. Consequently, immunohistochemical analysis was conducted on the tissue models to detect macrophages, specifically identifying antigens such as CD68 and CD206, which are indicative of the M1 and M2 phenotypes of macrophages, respectively (Hu et al., 2017; Peng et al., 2023). Our investigations yielded no evidence of the presence of macrophages in the tissue. Consequently, we hypothesise that communication between the primary fibro-blasts and macrophages occurs via the semi-permeable membrane, facilitated by soluble messengers. The impact of macrophages on cellular responses was substantiated by the disparate outcomes observed in the ELISA and IHC analyses, which revealed notable divergences between the M1 and M2 cultures.

#### 4.1.1 Macrophage differentiation and influence on fibroblasts

Differentiation of THP1 monocytes into M0, M1, and M2 macrophages was successfully achieved and characterized by specific markers. It is well established that macrophages exhibit distinct phenotypic and functional alterations in response to environmental stimuli, such as cytokines, pathogens, foreign particles, and tissue damage (Stout et al., 2005; Haschak et al., 2021). Consequently, it is imperative to refrain from the utilisation of donor macrophages within an *in vitro* systems, thereby ensuring the exclusion of any potential stimulation that may have been induced by as yet unidentified factors. The THP1 cells utilised in this study are influenced by stimulation and the various cytokines present in the microenvironment, undergoing a process of polarisation that subsequently leads to the differentiation of these cells into a variety of functional forms (Jannasch et al., 2017b) (3). Resting M0 macrophages can get polarized towards classic pro-inflammatory phenotype M1 macrophages by the stimulation with either microbial factors, like LPS, or using proinflammatory cytokines, such as IFN-γ. On the contrary, M0 can polarize towards M2 subset when exposed to cytokines such as IL-4, or IL-13 (38). The established SOP “THP1 - Differentiation” proved to be a reliable differentiation measure into the different macro-phage types (M1, M2, M0) and could be verified by immunofluorescence. The antibodies used against CD-markers served to monitor the success of differentiation. Anti-CD80 (Zhou et al., 2017) was used for M1 macrophages, anti-CD163 (Hu et al., 2017) for M2 macrophages and anti-CD68 (Wang et al., 2017) for M0 macrophages. Co-culture experiments demonstrated that the macrophage phenotype profoundly affects fibroblast behavior. M1 macrophages, typically associated with pro-inflammatory responses, induced higher levels of fibroblast activation and cytokine secretion compared to M2 macrophages, which are linked to anti-inflammatory and wound healing responses. This suggests that macrophage polarization can be a strategic target to modulate FBR and improve implant biocompatibility. We decided in favour of the cell line of THP1 cells for co-stimulation and against primary immune cells in order to exclude prior activation of the macrophages before removal from the donor organism, as this could have negative consequences on the usability of our measurements (Hörner et al., 2004; Borsini et al., 2020; Migulina et al., 2024).

#### 4.1.2 Dynamic remodeling in 3D collagen hydrogels

A substantial body of evidence from numerous studies has demonstrated that M2 macrophages exert a profound impact on collagen production, particularly through their capacity to modulate processes such as defect healing and the three-dimensional structure of collagen (Knipper et al., 2015). This is achieved by modulating the activity of fibroblasts, which are stimulated to form the extracellular matrix (ECM) via the TGF-β1 pathway (Yang et al., 2023). The objective of our study was to further investigate the capacity of these cells to regulate collagen. Initially, we focused on procollagen formation up to day 10 following CT800 stimulation. Subsequent experiments involving metal particles shifted the focus to collagen I production, extending the analysis to a 14-day period. It is well documented that procollagen, the precursor to collagen, is produced in large quantities at the onset of tissue regeneration or wound healing. Typically, production decreases over time as healing progresses (Birk, 2001; Gelse et al., 2003). In this con-text, the 3D collagen hydrogel model provided a physiologically relevant environment in which to study fibroblast behaviour over time. The embedded fibroblasts exhibited dynamic alterations in proliferation, procollagen and collagen I expression, which mirror their pivotal function in matrix remodelling. The results of the time-course analysis demonstrated that the responses of the fibroblasts varied significantly in accordance with the stimulation by particles. This underscores the importance of temporal studies in order to gain a deeper understanding of the long-term tissue integration processes and the potential for the mitigation of fibrosis. These insights are of critical importance for the design of biomaterials that not only promote favourable tissue responses but also enhance the longevity of implants. The results demonstrated that fibroblasts produced collagen I, which was used to construct the extracellular matrix, regardless of whether they were stimulated or not, and even in the absence of external signals. The production of collagen remained largely unaltered across different macrophage cultures, whether in the absence of stimuli or following stimulation with steel. However, following stimulation with titanium, notable differences were observed. The M2 macrophage culture demonstrated a considerable increase in collagen production, reaching approximately 150% of the baseline level over time, whereas the M1 culture exhibited a significant decline, with collagen production decreasing by approximately 50%. In conclusion, it can be stated that both procollagen and collagen I are suitable for the detection of ECM synthesis by fibroblasts and for the demonstration of the connection with different types of macrophages. Given that pro-collagen is strongly expressed at the initial stage of the FBR, with collagen I forming several days later, we propose measuring the proportion of procollagen in short-term cultures and collagen I in long-term cultures (>10 days).

### 4.2 Cytokine and chemokine mediated communications

#### 4.2.1 Specific role of cytokine for cell communication in biosystem

The regulation of FBR in the human body and in in vitro biosystems is a highly intricate and multifaceted set of rules (Anderson et al., 2008; Mariani et al., 2019). In this context, the primary objective of our experimental configuration was to ascertain consistent concentration patterns of cytokines that can be uniformly expressed, irrespective of the material composition of the individual stimulants. Concurrently, the number of pro- and anti-inflammatory cytokines investigated was limited, as the high number of different cytokines (Holdsworth and Gan, 2015) would not be suitable for standardising the measurement principle. This methodological approach facilitates a more precise and standardised analysis of immune responses, as the variability cannot be distorted by different materials or physical properties of the stimuli. Cytokines are optimal markers for the study of immune responses, as they can reflect the activation and modulation of immune cells in real time. A significant benefit of utilising ELISA for cytokine measurement as opposed to conventional methods, such as PCR or immunohistochemistry, is its capacity for non-invasive assessments (Jalalypour et al., 2016). Conventional methods necessitate the extraction of cells from their culture environment and subsequent fixation, a process that results in the cessation of culture and the determination of endpoint measurements. Additionally, there is a possibility that the RNA quantity determined by PCR does not accurately reflect the protein level (Amsen et al., 2009). Consequently, the continuous monitoring of reactions over time is hindered by PCR and IHC, as they necessitate the creation of multiple cultures. In contrast, cytokine measurements by ELISA can be performed directly from the culture medium without disturbing or terminating the cell culture. Furthermore, the samples can be analysed without complex pre-treatment (Sakamoto et al., 2018). This enables simple, continuous and dynamic monitoring of the immune response over time, facilitating a more precise assessment of alterations in cytokine concentrations and, consequently, the biological response to implants and other stimuli. This facilitates the early detection of changes in the inflammation and regeneration process. These advantages make measurement by ELISA an invaluable tool for monitoring cell responses in in vitro models, as it allows detailed and continuous observation of process dynamics without the need to interrupt the culture phase.

#### 4.2.2 Measurement of cytokine profiling

Both sandwich ELISA and multiplex ELISA were used for the qualitative and quantitative determination of cytokines. In principle, both methods are suitable for the accurate determination of cytokine concentrations in cell culture supernatants (Leng et al., 2008). In our experimental setup, we found that the available sample volume was quickly reached if the supernatant was collected regularly (on days 3, 7, 10, 14). In our work, we have aimed to identify a cytokine pattern that can give an indication of the tissue compatibility of a foreign material. For this reason, we believe that the multiplex ELISA method will be more practical in the future, as it requires less sample material for the determination of different cytokines and they can be determined simultaneously. In conclusion, the higher time and material efficiency of the multiplex method compared to the sandwich ELISA is an important factor for the future evaluation of clinical feasibility.

#### 4.2.3 Cytokine profiling

The cytokine profile measurements indicate that IL-6 and TGF-β1 are instrumental in the expression of the tissue response (Meléndez et al., 2010; Gingery et al., 2014; Sheikh et al., 2015). It is evident that the unstimulated culture demonstrates a lack of IL-6 expression, indicating that the cells require an external stimulus (FBR) to initiate the production of IL-6. In order to establish a connection between IL-6 formation and foreign body stimulation, a variety of materials were employed. The elevation in IL-6 levels over the 14-day observation period was evident across all materials tested. This demonstrated that IL-6 is an appropriate marker for examining whether a tissue model has been stimulated with a foreign body or whether a non-stimulated culture is present. As has been previously documented in the scientific literature, interleukin (IL-6) plays a role not only in the inflammatory response of the immune system, but also in the modulation of anti-inflammatory processes within the body (Fu et al., 2017; Borsini et al., 2020; Yan et al., 2023). This could explain why IL-6 is produced in greater quantities after stimulation with titanium than with steel, given that titanium is more biocompatible and therefore presumably elicits a stronger anti-inflammatory response (Hanawa, 2022). This theory is supported by the observation that the concentration of IL-6 is higher in the co-culture with the anti-inflammatory M2 macrophages. Furthermore, the increasing concentration of TGF-β1 over time (day 3 to day 14) following stimulation with titanium particles lends additional support to this hypothesis. Recent findings have demonstrated that IL-6 stimulates the production of TGF-β1 (Fu et al., 2017). Given that TGF-β1 is crucial for the promotion of tissue proliferation, the elevated concentration of this cytokine in the titanium-stimulated culture relative to the steel culture suggests enhanced tissue adaptation (Wahl et al., 1988). The concentration of IL-4 is higher in M2 macrophages than in M1 macrophages at the outset of the culture period. This is due to the fact that M2 macrophages are activated in an anti-inflammatory environment and produce IL-4, which is an important signaling substance that promotes repair processes and tissue healing (Mosser and Edwards, 2008). These cells initially demonstrate an augmented response to foreign body stimulation, as they utilise elevated IL-4 production during the initial phase to modulate inflammation and tissue repair (Dunster, 2016). However, as the culture progresses, there is a reduction in IL-4 levels as macrophages adapt to microenvironmental stimulation. Furthermore, the production of pro-inflammatory mediators decreases as tissue repair progresses. Therefore, the greatest degree of clarity was observed in the measured values of IL-4, IL-6 and TGF-β1. Conversely, no discernible differences or similarities were identified in the patterns of the other cytokines with regard to concentration level or tendency.

#### 4.2.4 Prominent cytokine factors or enzymes for FBR phases

Tumour necrosis factor-α (TNF-α) is a pro-inflammatory cytokine that is liberated during the early stages of inflammation, stimulated the recruitment of immune cells and the activation of inflammatory processes (Parameswaran and Patial, 2010). Macrophages that have been stimulated with particles favor to produce pro-inflammatory cytokines following interaction (Zhang and An, 2007). IL-6 has categorised as a classic pro-inflammatory cytokine for long time. However, newer studies have demonstrated that the concentration of IL-6 has an influence on the pro-inflammatory or anti-inflammatory function (Borsini et al., 2020). It has been demonstrated that an elevated concentration of IL-6 is conducive to an anti-inflammatory response and can inhibits the activity of M1 (Fu et al., 2017). Elevated levels of pro-inflammatory cytokines (IL-6, TNF-α) were observed in cultures with M1 macrophages, while M2 macrophages promoted anti-inflammatory cytokine production (IL-10, TGF-β1). These cytokine profiles offer predictive markers for the type of tissue response (inflammatory vs. regenerative) elicited by different implant materials, aiding in the design of biomaterials that promote favorable outcomes.

### 4.3 Numerical evaluation of the results

Two distinct approaches were available for the analysis and interpretation of the measurement results. Firstly, the comparison of absolute concentration values was conducted. Secondly, the changes in concentration as a percentage and the assessment of the trend were carried out. Our investigations revealed considerable discrepancies in the absolute concentration values, not only between the various stimulation materials but also within a given material group, as evidenced by the differences observed between individual measurements conducted using ELISA or IHC. In contrast, the tendencies exhibited a similar pattern. Accordingly, it seems prudent to evaluate the discernible trends of the cytokines and ECM components (procollagen, collagen I, and actin) rather than the absolute concentration values, with a view to assessing the FBR. This approach ensures greater comparability between the groups.

## 5 Conclusion

To develop an *in-vitro* assay to simulate foreign body reaction is a challenging task given the requirement of considerations of various factors, namely, biomimetic scaffold selection and *in vivo* ECM like resemblance, assessment methods and critical reference cytokine selection. In this study, we developed an *in vitro* assay for foreign body reaction using collagen hydrogel as a biomimetic 3D scaffold which presents a significant improvement over conventional tests such as patch testing (PT) and lymphocyte transformation test (LTT).

### 5.1 Clinical background

The study is of great importance as it advances our comprehension of the interactions between implant materials, macrophages and fibroblasts, which is pivotal for the advancement of more biocompatible implants. The data demonstrate that M2 macrophages, which elicit an anti-inflammatory response, facilitate collagen synthesis, particularly when stimulated with titanium, whereas M1 macrophages tend to inhibit this process. This is corroborated by the heightened expression of TGF-β1 in the M2 cultures, which points to a more pronounced promotion of fibrosis. Upon examination of cytokine production, a notable elevation in IL-6 was discerned in both M1 and M2 cultures following stimulation with titanium particles. This observation may suggest an intensified inflammatory response or provide evidence for differentiation of macrophages towards an anti-inflammatory phenotype (Fu et al., 2017). Furthermore, it was observed that collagen synthesis was significantly increased in co-cultures with M2 macrophages in comparison to M1 cultures, which suggests that matrix formation and tissue regeneration were more pronounced. A principal objective of the study was to develop a standardized *in vitro* method for estimating an implant response. In light of these findings, we emphasise that an assessment based on absolute numerical and concentration values is not considered meaningful. Instead, the evaluation of trends is of great importance, given that the culture was stimulated with completely different materials. The measurement of cytokines enables the assessment of the tissue reaction over time, thereby avoiding potential issues such as primary sensitisation or excited skin syndrome (ESS) that may arise in the context of the previously employed epicutaneous patch test (Garg et al., 2021). Furthermore, it should be noted that alterations in the results of the patch test may occur with repeated application (Duarte et al., 2002). Traditional patch tests suffer from false negatives, lack of long-term response assessment, and variability in results (Ophaug and Schwarzenberger, 2020; Rodriguez-Homs et al., 2020; Garg et al., 2021). More sophisticated variants like LTT tests are costly, time-consuming, and may lack sensitivity in detecting weak immune responses (Sittiwattanawong et al., 2024). We consider our method to be a suitable alternative, as the stimulation only occurs after the tissue cells have been removed from the donor organism, thereby protecting the patient from the potential adverse effects of the test. By utilizing a 3D biomimetic collagen hydrogel based scaffold, our assay enables an *in vitro* system to provide reproducible and cost effective alternative for preclinical biomaterial testing, reducing their reliance on unreliable animal or patient testing method. This in turn minimizes ethical concerns related to *in vivo* trials as well.

### 5.2 Future work

The further stages of this research could concentrate on modifying the stimulation of fibroblasts and macrophages in order to gain a deeper comprehension of their interactions and the underlying mechanisms. One potential avenue for further investigation would be to stimulate both fibroblasts and macrophages simultaneously, with a view to studying their coordinated response to implant materials. In order to eliminate the possibility of an immunological influence of an animal collagen matrix on tissue processes, a synthetic matrix similar to human collagen could be used in future research. For example, Vecollan**®** from Evonik, gelatin plus PEG hydrogel models can be explored. Furthermore, the utilisation of patient-specific models could facilitate a personalised investigation of tissue response to implants, thereby elucidating the variability of responses observed in patients who are allergic or not to implants. It is hypothesised that patients may demonstrate disparate trajectories in their response to implants, which could be reflected, for example, in the temporal dynamics of cytokine production. In some cases, the increase in cytokine production may be more rapid and pronounced, whereas in other patients, it may be delayed or less intense Additionally, discrepancies in the expression of collagen and other extracellular matrix components may also occur, which could consequently lead to disparate tissue healing processes and inflammatory reactions. In this context, a comparison of the tissue response of fibroblasts in patients who have responded to an implant with those who have not would be particularly informative. Such a comparison could provide valuable insights into the molecular and cellular differences that influence the success or failure of implantation. These approaches could ultimately contribute to the optimisation of biomaterials and the development of more precise, patient-specific therapeutic strategies that are tailored to individual immune responses and tissue reactions, with the aim of protecting patients.

## Data Availability

The raw data supporting the conclusions of this article will be made available by the authors, without undue reservation.
