# Supplementary figures and images for: Biomimetic hydrogel scaffolds for stimulating fibrotic responses: development of an in-vitro assay for implant material testing
Source: Front Bioeng Biotechnol. 2025 Aug 29;13:1628630. doi: 10.3389/fbioe.2025.1628630 (PMC12425956; doi:10.3389/fbioe.2025.1628630)

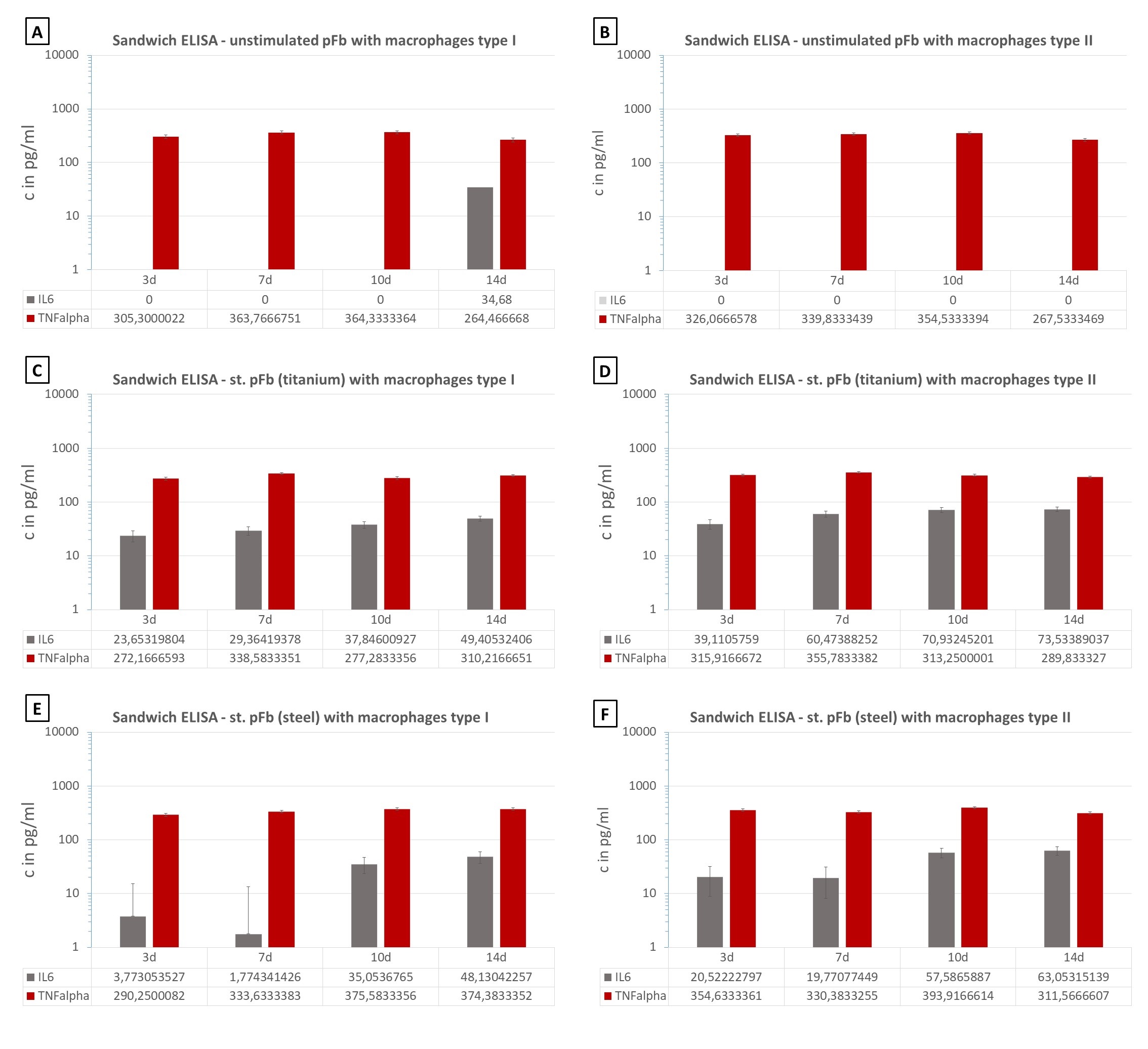

Supplement: Supplementary file 1 [file Image3.jpg]

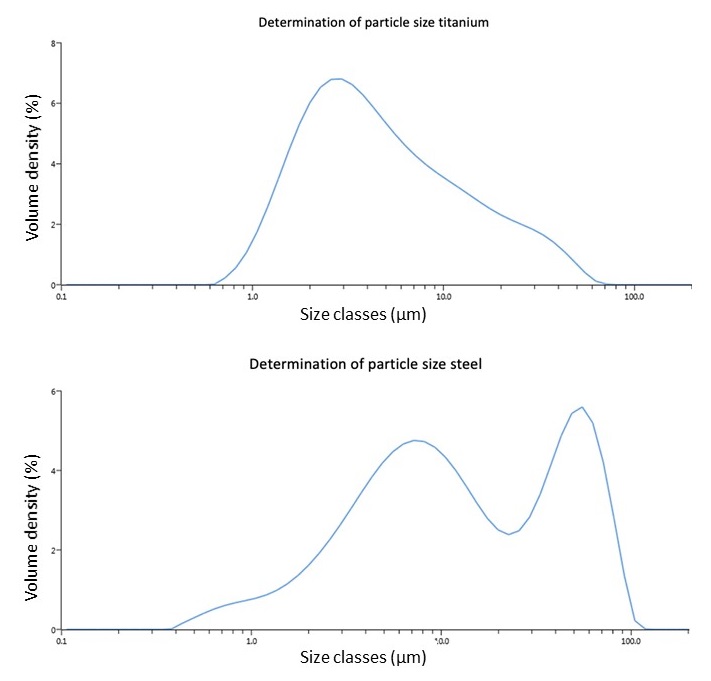

Supplement: Supplementary file 2 [file Image1.jpeg]

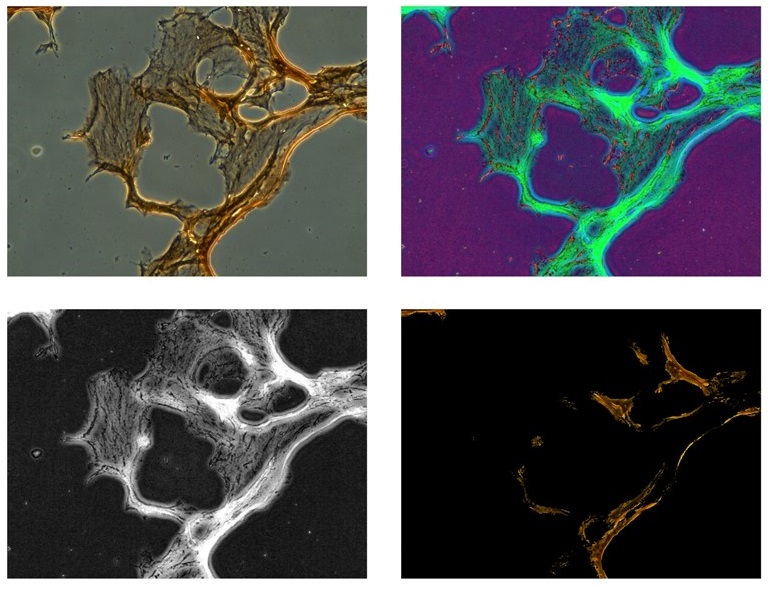

Supplement: Supplementary file 3 [file Image2.jpeg]

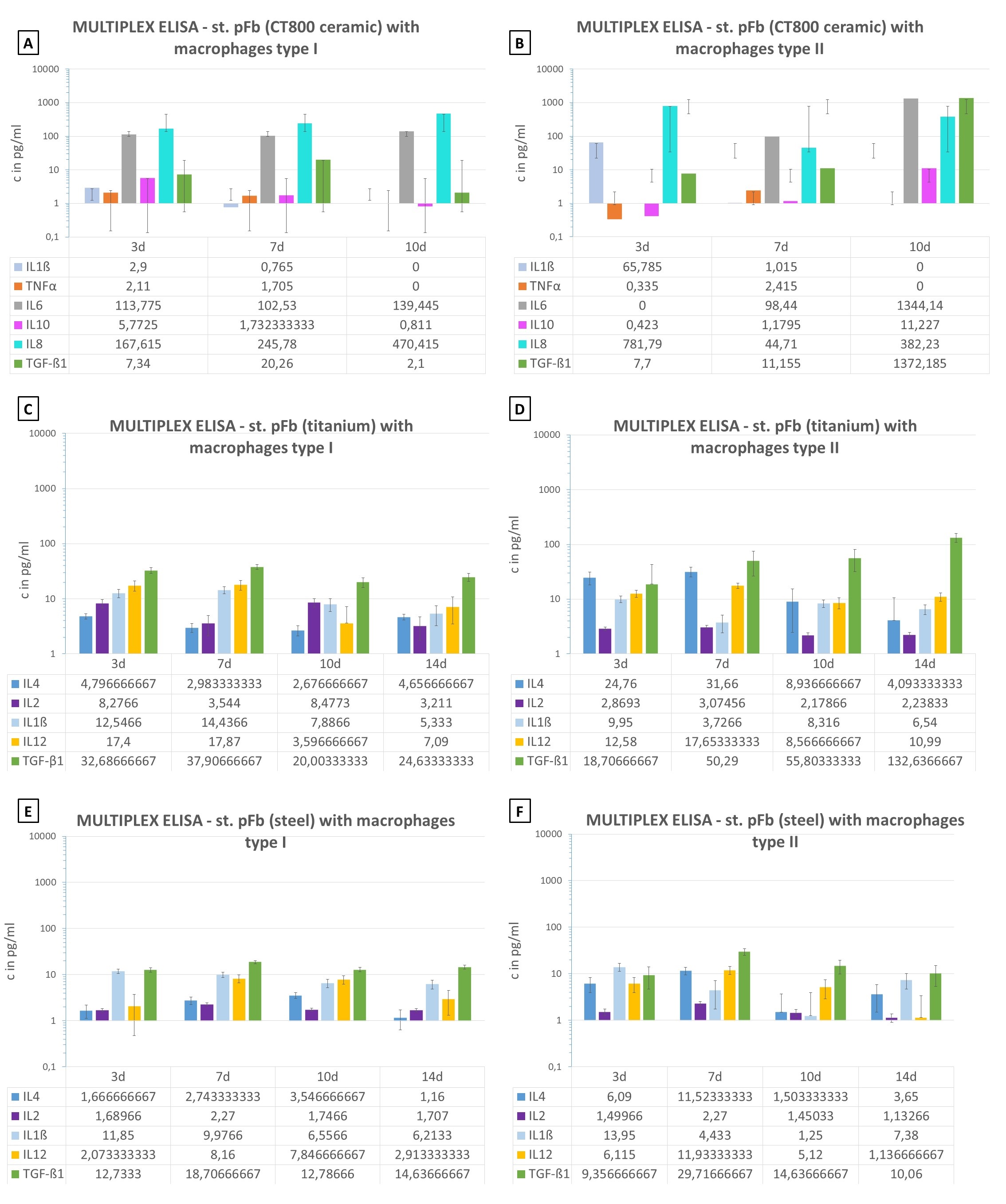

Supplement: Supplementary file 4 [file Image4.jpg]
